# Novel Long‐Acting Ghrelin Analogue PEP‐064 Restores Energy Balance in C26 and Lewis Lung Carcinoma–Induced cachexia in Mice

**DOI:** 10.1002/jcsm.70318

**Published:** 2026-06-01

**Authors:** J. E. Hunt, C. Lund, B. Chen, A. R. Diaz, O. Dmytriyeva, J. Marcotorchino, K. Löbner, K. Mörl, N. R. Andersen, Z. K. J. Ogueboule, E. Frank, J. Stöhr, D. Meseguer, V. Panajotova, J. Roux, C. Clemmensen, L. Sylow, M. Schneeberger, A. G. Beck‐Sickinger, S. L. Pedersen, K. Fosgerau

**Affiliations:** ^1^ Pephexia Therapeutics ApS Frederiksberg Denmark; ^2^ Department of Cellular and Molecular Physiology Yale University School of Medicine New Haven Connecticut USA; ^3^ Novo Nordisk Foundation Center for Basic Metabolic Research, Faculty of Health and Medical Sciences University of Copenhagen Copenhagen Denmark; ^4^ Biomeostasis La Penne sur Huveaune France; ^5^ Faculty of Life Sciences Leipzig University Leipzig Germany; ^6^ Department of Biomedical Sciences, Faculty of Medical and Health Sciences University of Copenhagen Copenhagen Denmark; ^7^ Apigenex sro Prague Czech Republic; ^8^ Wu Tsai Institute for Mind and Brain Yale University New Haven Connecticut USA

**Keywords:** C26 colon carcinoma, cancer cachexia, ghrelin, Lewis lung carcinoma, peptide

## Abstract

**Background:**

Cancer cachexia is a debilitating syndrome marked by involuntary weight loss resulting from reduced food intake and intricate metabolic reprogramming. Despite its high prevalence, cancer cachexia remains undertreated, with a lack of effective and approved pharmacotherapies. Ghrelin has emerged as a therapeutic target for cancer cachexia due to its beneficial effects on energy balance. However, the clinical application of ghrelin is hampered by its short half‐life. In this study, we introduce PEP‐064, a novel stabilized, long‐acting and efficacious ghrelin analogue and assess its effects on C26‐induced and Lewis lung carcinoma (LLC)–induced cachexia in mice.

**Methods:**

The in vivo efficacy of PEP‐064 was evaluated in healthy CD‐1 mice after repeated dosing for 7 days and in the C26 and LLC cancer cachexia mouse models. Additionally, the pharmacokinetic profile and whole brain neuronal activity mapping were conducted in healthy mice following a single subcutaneous injection of PEP‐064. Growth hormone secretion was measured in healthy rats following a single administration of PEP‐064.

**Results:**

In healthy mice, PEP‐064 exhibited a dose‐dependent (100, 300 and 1000 nmol/kg) increase in both delta body weight (BW) and total food intake (FI) compared to the vehicle (BW: 2.7 g vs. 3.5, 4.8 and 5.3 g; FI: 50 g vs. 57 g, 60 and 70 g). In the C26 model, PEP‐064 protected against loss of tumour‐free (TF) BW (−1.2 g vs. 1.7 g, *p* < 0.0001), increased food intake (33 g vs. 41 g, *p* < 0.01), prevented losses in fat (−1.1 g vs. 0.9 g, *p* < 0.0001) and lean mass (−0.07 g vs. 0.4 g, *p* < 0.05) without affecting tumour growth. In the LLC model, PEP‐064 induced hyperphagia (52.2 g vs. 61.6 g, *p* < 0.0001) and protected against TF‐BW loss (−1.4 g vs. − 0.01 g, *p* < 0.05) and fat mass loss (−0.9 g vs. 0.8 g, *p* < 0.0001). In mice, PEP‐064 had a T_1/2_ of 6.6 h and a *T*_*max*_ at 4 h. PEP‐064 increased neuronal activity (c‐Fos) in the hypothalamic and amygdala regions, with the tuberal nucleus of the hypothalamus showing the highest increase compared to the vehicle (*p* < 0.05). Lastly, circulating growth hormone was increased 20 min after subcutaneous PEP‐064 dosing, peaking at 30 min at 93 ng/mL and returning to approximately baseline by 120 min.

**Conclusions:**

The novel, long‐acting and efficacious ghrelin analogue, PEP‐064, restored energy balance in cancer cachexia by increasing food intake and body weight, preserving lean mass and increasing adiposity, without affecting tumour growth. Considering the unmet medical need for safe and effective treatments for cachexia, our study demonstrates the feasibility of a long‐acting ghrelin approach for treating cancer cachexia.

## 1 Introduction

Cancer cachexia is a debilitating syndrome marked by involuntary weight loss resulting from complex metabolic reprogramming associated with inflammation and anorexia [1]. Depending on the type of cancer, cachexia impacts 50%–80% of patients with advanced‐stage cancer [2] and accounts for up to 30% of cancer‐related deaths [1]. Patients suffer from severe wasting of both adipose tissue and skeletal muscle, leading to reduced quality of life (QoL), functional impairment and frailty. This deterioration further diminishes their tolerance to anticancer therapies and ultimately contributes to decreased survival [2].

Despite the high prevalence of cancer cachexia, the syndrome remains underdiagnosed and undertreated, with a lack of efficacious and approved drugs. Ghrelin, a pleiotropic peptide that increases during caloric restriction, has been proposed as a therapeutic target for cancer cachexia due to its impact on energy balance, mediated by increased appetite and body weight [3]. Ghrelin's influence on homeostatic food intake is mediated by its actions on the growth hormone secretagogue receptor (GHSR) upstream of the agouti‐related protein (AgRP)/pro‐opiomelanocortin (POMC) system, specifically activating orexigenic neurons that co‐express neuropeptide Y (NPY) and AgRP neurons, and the inhibition of POMC neurons in the hypothalamic arcuate nucleus (Arc) [4]. Additionally, Ghrelin's central nervous system distribution extends to the brainstem and limbic system, where it may influence food intake by modulating behaviours associated with reward, learning and memory [5]. Ghrelin also strongly stimulates growth hormone secretion from the anterior pituitary gland, which in turn regulates Insulin‐like Growth Factor (IGF)‐1 levels [3]. Given IGF‐1's role in promoting protein synthesis and muscle growth, ghrelin could potentially also benefit in mitigating the muscle wasting associated with cancer cachexia via this pathway.

In preclinical cancer cachexia studies, ghrelin has been shown to enhance food intake and body weight, decrease fat and lean muscle mass losses and increase overall survival [6, 7]. Furthermore, clinical trials involving cancer patients treated with native human ghrelin demonstrate promising food intake and QoL improvements without adverse effects or increased tumour progression [8, 9]. Despite these advances, the pharmacological potential of native ghrelin is limited by its short elimination half‐life (~11 min) [10] and rapid inactivation/deacylation in circulation, highlighting the need for new stabilized, long‐acting ghrelin analogues with improved pharmacokinetics.

Several small‐molecule ghrelin mimetics/GHSR agonists have been developed. Among these, anamorelin advanced to Phase 3 clinical trials, where it demonstrated effectiveness in increasing food intake, body weight, lean body mass, QoL and ECOG (Eastern Cooperative Oncology Group) performance status scale in patients with non‐small cell lung cancer (NSCLC). However, anamorelin failed to produce a significant improvement in hand grip strength, which ultimately contributed to its rejection by the FDA/EMA [11, 12]. Although anamorelin highlighted the potential of ghrelin‐targeted therapy, it also diminished enthusiasm for the ghrelin receptor as a therapeutic target and revealed limitations inherent to small‐molecule approaches, including off‐target effects and limited receptor specificity [13]. In contrast, peptide therapeutics offer highly selective receptor engagement, translating into improved safety, tolerability and efficacy profiles in humans [14]. However, their primary limitation is a short circulating half‐life, which can be addressed by designing stabilized analogues. In one experimental approach, a derivative of vitamin D, EXT418, was covalently conjugated to a modified ghrelin peptide for half‐life extension [15]. This compound was tested in an aged LLC model of cancer cachexia, where treatment led to increased food intake, body weight and preservation of both fat and lean mass. Beyond EXT418, prior literature describing long‐acting peptide ghrelin analogues is limited, though several patents exist (WO2013113916 and WO2015197037). Our approach utilizes well‐characterized strategies, with proven safety records in other clinical applications, to achieve lipidation and backbone stabilization of the native ghrelin peptide, resulting in a long‐acting, efficacious peptide.

Here, we demonstrate the feasibility and efficacy of using lipidation for peptide stabilization to provide a half‐life extended and constitutively active ghrelin analogue for treating cancer cachexia. We show PEP‐064 to be efficacious in increasing both body weight and food intake in healthy mice, demonstrate PEP‐064's ability to regulate hypothalamic neuronal activity and influence peripheral GH secretion and show that PEP‐064 restores energy balance in mouse models of cancer cachexia by inducing hyperphagia, reducing body weight loss and protecting from fat and lean mass losses, without affecting tumour growth.

## 2 Methods

### 2.1 Animals

All experiments were approved by either the Ministère de l'Enseignement supérieur et de la Recherche, France; the Ministry of Industry and Trade, Czech Republic; the Danish Animal Experimental Inspectorate; or the Institutional Animal Care and Use Committee of Yale University School of Medicine, which is consistent with the National Institutes of Health, United States, guidelines. All mice were single‐housed and maintained at a constant temperature (22°C ± 2°C) with fixed 12:12 h light–dark cycles and free access to water and standard chow diet. NMRI (Crl:NMRI/Han), CD‐1, BALB/c and C57BL/6J mice were purchased from Charles River or Jackson Laboratories and were acclimatized for 1 week before starting experiments. Sprague Dawley (Crl:CD) rats were purchased from Charles River and housed under standard conditions, two to three rats per cage, and acclimatized for 14 days before starting experiments.

### 2.2 Compounds

Human ghrelin, sequence H‐GS[S(O‐Oct)]FLSPEHQRVQQRKESKKPPAKLQPR‐OH, and PEP‐064, sequence H‐PS[Dpr(N‐Oct)]FLSPEHQRVQQRKESKKPPAKLQPR[K([C18DA][yE][OEG][OEG]‐)]‐NH_2_, were both synthesized as TFA salt by standard Fmoc‐based SPPS (solid‐phase peptide synthesis) at Wuxi AppTec, China. Dpr refers to L‐2,3‐diaminopropionic acid (3‐amino‐L‐alanine), Oct refers to octanoic acid, OEG refers to 2‐(2‐aminoethoxy)ethoxy]acetic acid, C18DA refers to octadecanedioic acid and yE refers to y‐glutamic acid (gamma‐glutamic acid). The glycine (G) in Position 1 and the octanoylated amino acid serine (S(O‐Oct)) in Position 3 of hGHR have been substituted with proline (P) and the octanoylated amino acid Dpr (Dpr(N‐Oct)), respectively, to improve metabolic stability. A lipidated lysine has been introduced in the C‐terminal (Position 29) to improve the in vivo half‐life.

Anamorelin·HCl was purchased at MedChemExpress (Cat. No. HY‐14734A). The dose of anamorelin used was 30 mg/kg (p.o.), equivalent to 51 440 nmol/kg, which is the standard dose used in several preclinical studies of cancer cachexia with beneficial effects [16].

### 2.3 PEP‐064 In Vitro Potency and Pharmacokinetics

Cell‐based GHSR‐1α activity was investigated in HEK293 cells (DSMZ ACC 305, authenticated by short tandem repeat‐profiling), cultured in DMEM/Ham's F12 (1:1, v/v) supplemented with 15% (v/v) FCS under humidified atmosphere at 37°C and 5% CO_2_. Cells were transiently transfected with mouse or human GHSR‐1α cDNA, C‐terminally fused to EYFP, in pVitro2‐hygro‐mcs (InvivoGen) using Metafectene (Biontex). The ligand potency was measured using the IP‐One HTRF assay system according to the manufacturer's instructions (Cisbio) in technical triplicate, *n* = 2 for PEP‐064 and *n* = 10 for the endogenous ligand ghrelin. Ligand dilution and assay were performed in HBSS with 20‐mM LiCl. The HTRF ratio was calculated, and EC_50_ and pEC_50_ ± SEM values were examined by nonlinear regression analysis.

The plasma concentration of PEP‐064 was investigated in four CD‐1 mice after a single subcutaneous injection (s.c.) (300 nmol/kg). Blood was collected from the saphenous vein at time points 0.5, 1, 2, 4, 8 and 24 h. The samples were prepared by mixing 30 μL of plasma sample with 60 μL of internal standard solution (100 ng/mL of Leucine‐enkephaline in acetonitrile with 1% of formic acid). The samples were then mixed for 3 min (1000 rpm), then centrifuged for 20 min at 2700×*g* and submitted to LC‐MS/MS analysis. The standard samples were prepared into mouse plasma by spiking the matrix into concentrations 1–10 000 ng/mL of the analytes, respectively, and otherwise treated as the samples.

### 2.4 PEP‐064 Pharmacology Profile In Vivo

On Day 0, 40 male (Crl:NMRI/Han) mice 22–24 g, received a once daily s.c. injection of either vehicle (0.9% NaCl in water with 0.01% ascorbic acid, pH = 4), human ghrelin (1000 nmol/kg), PEP‐064 (100 nmol/kg), PEP‐064 (300 nmol/kg) or PEP‐064 (1000 nmol/kg) for 7 days, *n* = 8 per group. Body weight was recorded each morning (8 AM) and evening (7 PM), and individual food intake was determined by subtracting the daily residual diet from the amount supplied. At termination, mice were anaesthetized with isoflurane, and blood was taken from the orbital venous plexus. Blood samples were stored in EDTA‐coated tubes, centrifuged (4 min, 10 000×*g*, 4°C) and stored at −80°C until exposure analysis. Subsequently, mice were terminated by cervical dislocation, and the muscles (m. gastrocnemius and m. soleus) and both epididymal and perirenal fat pads were separated and weighed.

Exposure analysis was measured in plasma samples by LC‐MS/MS. Briefly, the samples were prepared by mixing with internal standard solution (100 ng/mL of leucine‐enkephaline in acetonitrile with 1% of formic acid + 2% DMSO) for 3 min (1000 rpm), followed by centrifugation (20 min, 2700×*g*, 4°C). Standards were prepared in NMRI mouse plasma and spiked into concentrations of 1–10 000 ng/mL. Quality control samples were also prepared in NMRI mouse plasma and spiked into concentrations of 3, 30, 300 and 3000 ng/mL.

### 2.5 C26 Colon Carcinoma Mouse Model of Cancer Cachexia

Colon26 (C26) adenocarcinoma cells (Cytion, #400156) were cultured in RPMI 1640 medium (Gibco, #11875093, United States) supplemented with 10% foetal bovine serum (FBS, Sigma‐Aldrich, #F0804, United States) and 1% penicillin–streptomycin‐amphotericin B (ThermoFisher Scientific, #15240062, United States) (5% CO_2_, 37°C). Prior to inoculation, C26 cells were trypsinized and washed twice with Dulbecco's phosphate‐buffered saline (DPBS). C26 cells were centrifuged at 1600*g* for 3 min and suspended in 1:1 Matrigel:DPBS (ice cold) at a final concentration of 5 × 10^6^ cells/mL. All BALB/c mice were shaved on the right flank 1 day prior to the inoculation and randomized into experimental groups by body weight. Each mouse (age 10 weeks) received a s.c. injection with PBS with or without 5 × 10^5^ C26 cells into the right flank. After 5 days, the tumours became palpable and could be measured with callipers every second day. From Day 5, mice received either a s.c. treatment of vehicle (*n* = 12 and *n* = 8 in NTB) or PEP‐064 (1000 nmol/kg, *n* = 12) until the end of the study. Food intake, which was calculated as the difference in food hopper weight, was recorded daily alongside body weight. Mice developing ulcerations > 5 mm in diameter and tumours > 14 mm in average length and width (humane endpoint) were euthanized by cervical dislocation and excluded from the study according to the guidelines from the Danish Animal Experimental Inspectorate.

Total fat and lean body mass were measured 7 days prior to C26 inoculation by nuclear magnetic resonance using an EchoMRI (United States). A second MRI scan was conducted on Day 8, just before dissection. The final lean mass of C26 mice was determined by subtracting the tumour weight from the lean mass recorded before dissection.

### 2.6 Western Blot

Soleus muscle lysates were prepared from pulverized tissue homogenized in modified GSK3 buffer using a TissueLyser II. The supernatant was collected, and protein concentration was determined using the bicinchoninic acid method. Bovine serum albumin (BSA) was used as a standard (Pierce). Proteins were transferred to polyvinylidene difluoride membranes, blocked in TBS‐Tween containing 2% skim milk or 3% BSA and incubated overnight with primary antibodies at 4°C. After washing, membranes were incubated with horseradish peroxidase–conjugated secondary antibodies (1:5000). Bands were visualized using enhanced chemiluminescence and a ChemiDoc MP imaging system and quantified using Coomassie staining as a loading control. Details are described in the Supporting Information.

### 2.7 Haematoxylin and Eosin (H&E) and Cross‐Sectional Area Measurements

Muscle biopsies were embedded in optimal cutting temperature (OCT) compound and cryosectioned at a thickness of 8 μm using a cryostat maintained at −20°C, with muscle fibres oriented perpendicular to the blade. Three sections per animal were stained using H&E staining kit (ab245880, Abcam) according to the manufacturer's protocol. Imaging was performed using a Carl Zeiss AxioObserver microscope equipped with an EC Plab‐NEOFLUAR 10×/0.3 objective. Muscle fibre cross‐sectional area was quantified on whole‐section images using the Cellpose extension in QuPath [17] based on H&E staining profiles. Representative images found in Figure S1.

### 2.8 Lewis Lung Carcinoma (LLC) Mouse Model of Cancer Cachexia

Murine LLC cells were cultured in Dulbecco's modified Eagle's minimal medium supplemented with 10% FBS, 1% penicillin–streptomycin (5% CO_2_, 37°C). Before inoculation, cells were resuspended in sterilized PBS. C57BL/6J mice (age 6 weeks) were inoculated with 2 × 10^6^ of LLC cells (Groups 2–4) or an equal volume of heat‐killed LLC cells serving as a sham control into the left upper flank. After 5 days, the tumours became palpable and could be measured with callipers every second day. On Day 5, mice had their body composition analysed using a Minispec Analyser (LF50, Bruker, Germany) and began to receive treatment according to the following groups: (1) vehicle, s.c., twice daily, + vehicle, p.o., once daily, *n* = 8 (Sham—control); (2) vehicle, s.c., twice daily, + vehicle, p.o., once daily, *n* = 12 (LLC + vehicle); (3) vehicle, s.c., twice daily + anamorelin (30 mg/kg), p.o., once daily, *n* = 12 (LLC + Anamorelin); and (4) PEP‐064 (3000 nmol/kg), s.c., twice daily + vehicle, p.o., once daily, *n* = 12 (LLC + PEP‐064). Food intake, which was calculated as the difference in food hopper weight, was recorded daily alongside body weight. On Day 19, body composition was remeasured before termination. The study conformed to the humane endpoints of weight loss of over 20% body weight, 24‐h anorexia or a high distress score.

Grip strength was assessed on Days 5 and 19. Mice were placed on the grip metre (Bioseb) with their forelimbs and hindlimbs on the grid. Once on the apparatus, traction was applied via the tail. The resistance exerted by each mouse on the grid was recorded, with three trials conducted per mouse. Average grip strength and max grip strength were analysed.

### 2.9 Whole‐Brain c‐Fos Imaging

C57BL/6J mice (*n* = 3–4) were treated with a single s.c. injection of vehicle or PEP‐064 (1000 nmol/kg) and euthanized with a rising gradient of CO_2_ and intracardially perfused with phosphate buffered saline (PBS) followed by 4% paraformaldehyde (PFA). Whole brain immunolabelling for c‐Fos (rabbit polyclonal anti‐cFos, Synaptic Systems 226003 at 1:2000) was performed following the iDISCO+ protocol previously described [18] with slight modifications. All the steps were executed at room temperature with gentle nutating shaking unless otherwise specified. All the buffers were supplemented with 0.01% sodium azide (Sigma‐Aldrich) to prevent bacterial and fungal growth. After immunostaining, the samples were washed in PBS‐T (twice for 1 h and then overnight), dehydrated in a methanol/water increasing concentration series (20%, 40%, 60%, 80% and 100%, 1 h each and finally methanol 100% overnight), followed by a wash in 33% methanol in dichloromethane for 3 h. Methanol was washed out with two final washes in dichloromethane 100% (15 min each), and finally, the samples were cleared and stored in dibenzyl ether (Sigma‐Aldrich) until light‐sheet imaging. The acquisitions were done on a LaVision Blaze microscope equipped with infinity‐corrected objectives using the Alexa 488 (autofluorecence) and 647 (immunolabelling) filters. The brain was positioned in sagittal orientation, cortex side facing the light sheet, to maximize image quality and consistency. Analysis was conducted using ClearMap according to iDISCO+ protocols [18]. In brief, tiled acquisitions of Fos‐immunolabelled iDISCO+ cleared brains scanned with the light sheet microscope were processed with ClearMap2 [18] to generate both voxel maps of Fos cell densities, as well as region‐based statistics of cell counts. The stitching was done with Wobbly Stitcher the cell detection was run with parameters in ClearMap2. The alignment of the brain to the Allen Brain Atlas was based on the acquired autofluorescence image using Elastix. Filtered cell's coordinates were transformed to their reference coordinate in the Allen Brain Atlas common coordinate system. For voxel maps, spheres of 375‐μm diameter were drawn on each filtered cell. *p*‐value maps of significant differences between groups were generated using Mann–Whitney *U* test (SciPy implementation). Aligned voxelized datasets from each group of animals were manually inspected to identify the regional overlaps of *p*‐value clusters.

### 2.10 Growth Hormone Rat Secretion Model

Male Sprague Dawley (Crl:CD) rats, 226–250 g upon arrival (Charles River, Germany), were divided into three treatment groups; (1) vehicle, s.c., *n* = 8; (2) ghrelin (1000 nmol/kg), s.c. *n* = 10; and (3) PEP‐064 (3000 nmol/kg), s.c., *n* = 4. Each rat was dosed once (10 AM), and 200‐μL blood was sampled from the tail vein at Minutes −5, 10, 20, 30, 60, 90 and 120 and collected into Microtainer Microgard Li‐heparin tubes (Becton Dickinson, United States). The blood was centrifuged (4 min, 10 000×*g*, 4°C), and plasma was stored at −80°C until using the rat/mouse growth hormone ELISA kit (BioVendor, Czech Republic, Cat. Number RMEE023R).

### 2.11 Statistics

All data processing and graph generation were performed using GraphPad Prism Version 9, unless specified (GraphPad Software Inc., United States). For single‐variable comparisons, a one‐way ANOVA was utilized, followed by a post hoc Dunnett multiple comparison test against the vehicle group. For comparisons involving two or more variables, a two‐way ANOVA was employed, followed by a post hoc Tukey multiple comparison test against the vehicle group.

The area under the curve was used to compare GH data, and an unpaired *t* test was used to compare neuronal activity between treatment and vehicle. Results are presented as mean ± SEM and differences with *p* < 0.05 were considered significant.

### 2.12 Generative AI and AI‐Assisted Technologies

During the preparation of this work, the authors used ChatGPT 5.5 in order to improve readability and language. After using this tool, the authors reviewed and edited the content as needed and take full responsibility for the content of the published article.

## 3 Results

### 3.1 PEP‐064 Increases Food Intake and Body Weight in Healthy Mice

For the feasibility of PEP‐064 as a cancer cachexia treatment, we first evaluated its in vitro potency, pharmacokinetic profile and efficacy in healthy mice. The in vitro potency was investigated both towards the murine and human receptors, showing nanomolar potency. At the murine receptor PEP‐064 has a 15‐fold lower potency than native human ghrelin, EC_50_ 52.7 nM, *n* = 2 versus 3.6 nM, *n* = 10 (Figure S2). Similarly, at the human receptor, PEP‐064 had a 14‐fold lower potency than native human ghrelin, EC_50_, 49.4 nM, *n* = 2 versus 3.6 nM, *n* = 10 (Figure S2). The pharmacokinetic (PK) evaluation of PEP‐064 (300 nmol/kg, s.c.) in mice showed a *C*_*max*_ of 3750 μg/mL at 4 h (*T*_*max*_) and an elimination half‐life of approximately 6.6 h (Figure S2). Using allometric PK scaling will support once‐weekly dosing in humans.

**FIGURE 1:**
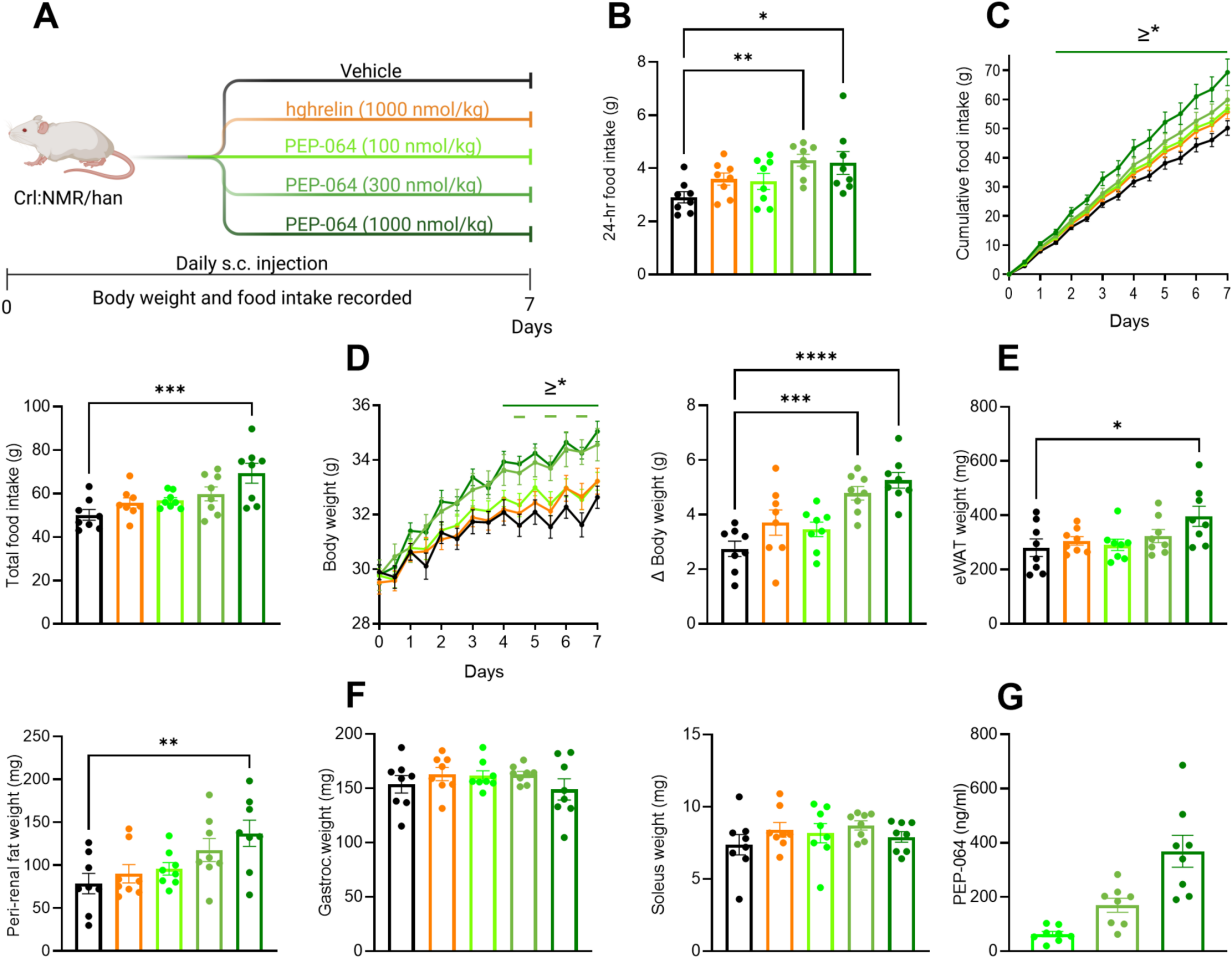
PEP‐064 pharmacology profile in mice. (A) Study outline. (B) 24‐h food intake. (C) Cumulative food intake and total food intake. (D) Body weight and change in body weight. (E) Epididymal (eWAT) and perirenal fat weight. (F) Gastrocnemius and soleus muscle weights. (G) Plasma PEP‐064 on Day 7. Data are shown as mean with SEM, *n* = 8. *p < 0.05, **p < 0.01, ***p < 0.001 and ****p < 0.0001 by either two‐way ANOVA or one‐way ANOVA compared to vehicle.

The in vivo efficacy of PEP‐064 on food intake and body weight was assessed following daily s.c. injections at three doses for 7 days in healthy mice (Figure 1A). Within the first 24 h, PEP‐064 treatment increased food intake in the two highest treatment groups (300 and 1000 nmol/kg) compared to the vehicle group, whereas 24‐h food intake in the lowest (100 nmol/kg) and the native human ghrelin (hghrelin) treatment groups was not affected (Figure 1B). Treatment with PEP‐064 dose‐dependently increased cumulative and final total food intake throughout the study (Figure 1C). However, only the highest dose of PEP‐064 (1000 nmol/kg) reached statistical significance compared to the vehicle group. In accordance with the hyperphagic response, treatment with PEP‐064 dose‐dependently increased body weight (Figure 1D). These effects were more pronounced after Day 4 of treatment in both of the groups treated with PEP‐064 (1000 nmol/kg) and during the evening weighing of PEP‐064 (300 nmol/kg) treated animals. This resulted in a marked change in body weight from Days 0 to 7, with mice treated with PEP‐064 (300 and 1000 nmol/kg) showing a positive weight gain (Figure 1D). In contrast, hghrelin did not influence body weight (Figure 1D).

Next, to assess the impact of PEP‐064 on fat and muscle mass, representative samples were dissected and weighed. PEP‐064 at 1000 nmol/kg increased both epididymal and perirenal fat mass when compared to vehicle‐treated animals (Figure 1E). In healthy mice, neither PEP‐064 nor hghrelin treatment affected the measured muscle weights (Figure 1F). On Day 7, plasma levels of PEP‐064 were quantified, revealing an incremental and dose‐dependent increase in PEP‐064 detected in plasma (Figure 1G). Together, these findings support the feasibility of PEP‐064 as a treatment for cancer cachexia, demonstrating its beneficial effects on energy balance by enhancing food intake, increasing body weight and promoting fat accumulation.

### 3.2 PEP‐064 Treatment Improves Body Weight Loss and Food Intake and Prevents Fat Mass and Lean Mass Losses in the C26 Colon Carcinoma‐Induced Model of Cancer Cachexia

**FIGURE 2:**
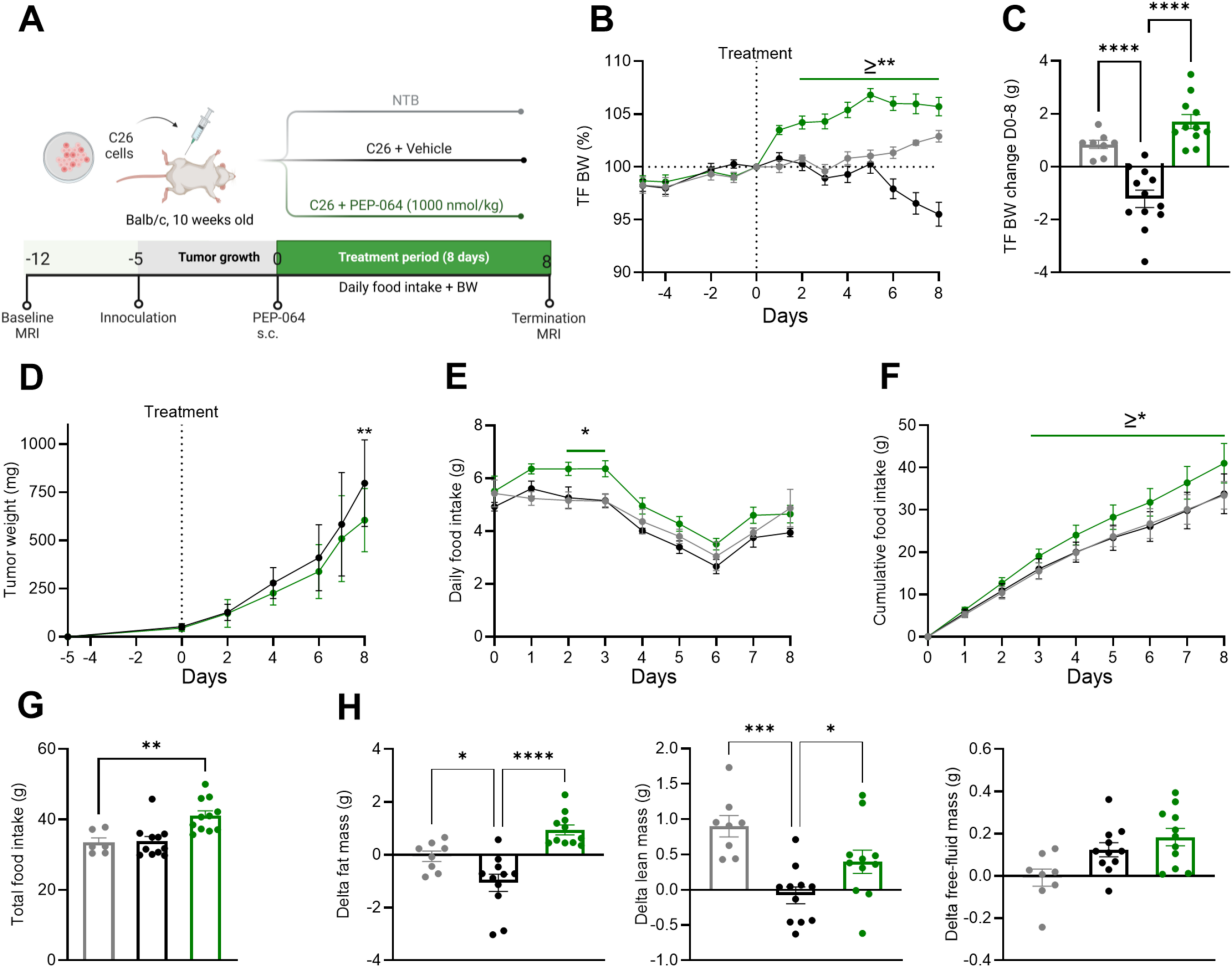
PEP‐064 ameliorated body weight loss, increased food intake and preserved fat and lean mass in the C26 colon carcinoma mouse model of cancer cachexia. (A) Study outline. (B) Tumour‐free body weight expressed as percent body weight measured on Day 0 (start of treatment). (C) Tumour‐free body weight change during treatment. (D) Daily tumour volume approximations. (E) Daily food intake, (F) cumulative food intake and (G) total food intake. (H) Body composition analysis: including change in fat, lean and free‐fluid mass. Data are shown as mean with SEM, *n* = 8–11. *p < 0.05, **p < 0.01, ***p < 0.001 and ****p < 0.0001 by one‐way ANOVA or two‐way ANOVA compared to C26 + vehicle.

Next, we evaluated PEP‐064's potential to mitigate cancer cachexia in the C26 colon carcinoma mouse model (Figure 2A). PEP‐064 (1000 nmol/kg/day) significantly increased tumour‐free body weight from Day 2 onwards and mitigated the rapid body weight decline observed from Day 5 in the cachectic C26 vehicle treated group (Figure 2B). At termination, despite C26 inoculation, PEP‐064 treated mice exhibited an average body weight increase of 1.7 ± 0.2 g, surpassing that of non‐tumour‐bearing mice (Figure 2C). In contrast, vehicle‐treated C26 mice experienced an average weight loss of −1.2 g ± 0.3 g, equating to a −5% tumour‐free body weight loss. Tumour weight was similar between the two tumour‐bearing groups until the termination day, when the vehicle treated tumours began to grow exponentially, prompting the termination of the study due to humane endpoints regarding tumour size (Figure 2D). Despite the absence of anorexia in the model, PEP‐064 increased both daily and cumulative food intake, resulting in a 22% or 7.62‐g increase in total food intake compared to the C26 vehicle treated animals (Figure 2E–G). Body composition assessed by MRI showed that PEP‐064 not only protected against C26‐induced fat mass losses but also increased fat mass above that of the non‐tumour‐bearing mice, *p* = 0.012 (Figure 2H). PEP‐064 also protected from lean mass losses seen in the model (Figure 2H). Free fluid mass tended to increase after tumour inoculation (*p* = 0.056), with no significant effect of PEP‐06 (Figure 2H).

**FIGURE 3:**
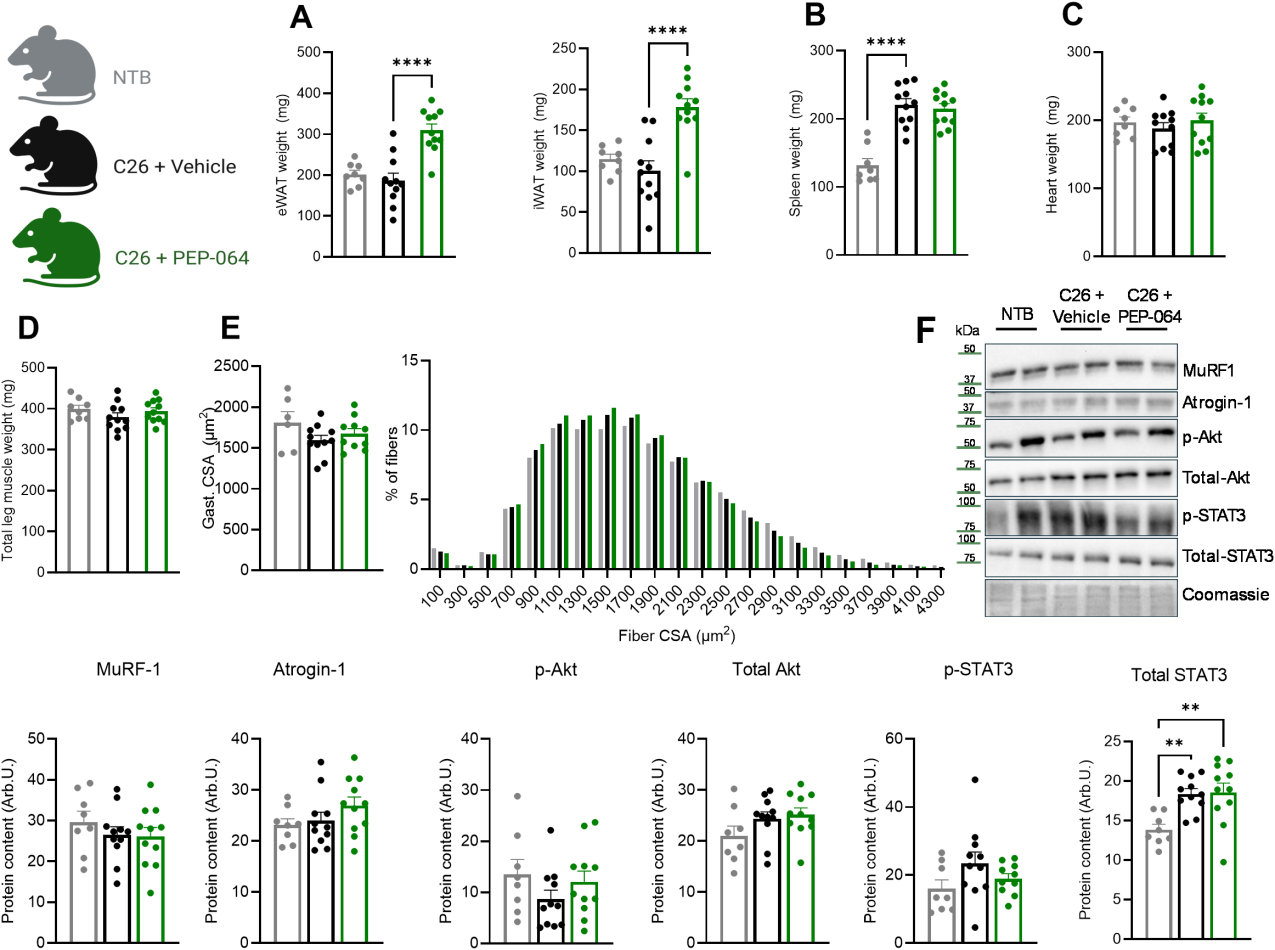
PEP‐064 treatment increased fat depot weight in the C26 colon carcinoma mouse model of cancer cachexia. (A) eWAT and iWAT depot weights. (B) Spleen and (C) heart weight. (D) Total leg muscle weight (sum of soleus, gastrocnemius, tibialis anterior, extensor digitorum longus and quadriceps). (E) Cross‐sectional area (CSA) of gastrocnemius muscle fibre and frequency plot of fibre CSA distributions. (F) Western blot analysis MuRF‐1, atrogin‐1, phospho‐Akt (p‐Akt), total Akt, phospho‐STAT3 (p‐STAT3) and total STAT3 in whole‐soleus lysate. Data are shown as mean with SEM, *n* = 8–11. **p < 0.01 and ****p < 0.0001 by one‐way ANOVA compared to C26 + vehicle.

Representative visceral (eWAT) and subcutaneous (iWAT) fat depots were dissected and weighed. For both fat types, C26 inoculation slightly reduced the fat mass, but the differences were not significant (Figure 3A). In contrast, PEP‐064 treatment not only preserved both visceral and subcutaneous fat but also increased the weight of the depots beyond that observed in non‐tumour‐bearing mice. The spleen was similarly enlarged in tumour‐bearing groups (Figure 3B). Heart weight was unaffected by C26 inoculation or treatment (Figure 3C). Despite a general trend for muscle weights to be reduced by C26 inoculation (Figure S3), total leg muscle weight was unaffected by C26 inoculation (Figure 3D). Gastrocnemius cross‐sectional area (CSA) was unchanged between the groups; however, there was a shift for a higher percentage of smaller fibres in the C26‐innoculated animals compared to non‐tumour‐bearing mice (Figure 3E). The atrogenes MuRF‐1 and atrogin‐1 were unchanged in the soleus (Figure 3F). Akt signalling showed a non‐significant trend, with phosphorylated Akt slightly decreased and total AkT slightly increased in the C26‐innoculated animals (Figure 3F). STAT3 protein levels were increased in both C26‐inoculated groups, but this was not reflected in the levels of phosphorylated STAT3 (Figure 3F).

Together, these data demonstrate PEP‐064's potential to restore energy balance in cancer cachexia by inducing hyperphagia, reducing body weight loss, preserving fat and lean mass and increasing adiposity, without affecting tumour growth.

### 3.3 PEP‐064 Treatment Ameliorates Body Weight Loss, Improves Food Intake and Prevents Fat Mass Loss in the LLC‐Induced Model of Cancer Cachexia

**FIGURE 4:**
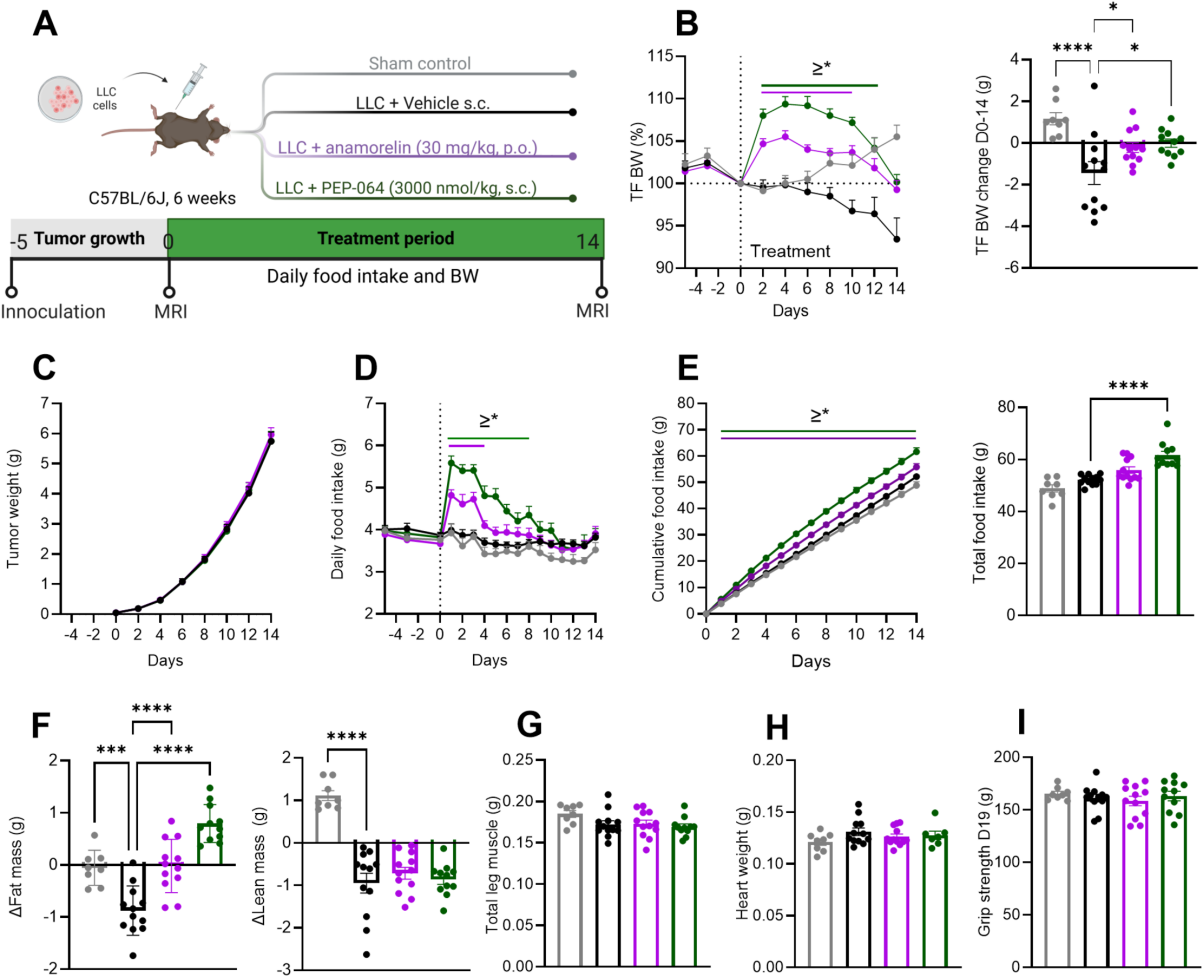
PEP‐064 treatment ameliorated body weight loss, increased food intake and preserved fat mass in the LLC mouse model of cancer cachexia. (A) Study outline. (B) Tumour‐free body weight expressed as percentage body weight measured on Day 0 (start of treatment) and tumour‐free body weight change during treatment. (C) Daily tumour volume approximations. (D) Daily food intake, (E) cumulative food intake and total food intake. (F) Body composition analysis including change in fat and lean mass. (G) Total leg muscle weight, sum of soleus, gastrocnemius, extensor digitorum longus and tibialis anterior. (H) Heart weight. (I) Average grip strength (mean of three trials) measured on Day 19. Data are shown as mean with SEM, *n* = 8–12. *p < 0.05, ***p < 0.001 and ****p < 0.0001 by one‐way ANOVA or Two‐way ANOVA compared to LLC + vehicle.

In a second cachexia model, the LLC mouse model (Figure 4A), PEP‐064 treatment increased tumour‐free body weight from Days 2 to 12 compared to the vehicle treatment (Figure 4B). Despite the clear positive effect on body weight, PEP‐064 treatment did not prevent LLC‐induced late‐stage body weight loss in the 2 days preceding the end of the experiment. However, PEP‐064 still ameliorated the overall body weight loss measured on Day 14, showing −0.01 ± 0.19 g versus −1.44 ± 0.54 g (Figure 4B). An anamorelin treatment group was included as a positive control, demonstrating similar protective effects against body weight loss. The changes in body weight in both treatment groups did not affect tumour weight (Figure 4C).

Surprisingly, LLC inoculation did not induce anorexia, even in the later stages of the model with a high tumour burden (Figure 4D). Despite this, PEP‐064 increased daily food intake from Days 1 to 8; the effects were most potent after the first exposure, increasing food intake from approximately 3.6 to 5.5 g (Figure 4D). However, daily food intake returned to baseline around Day 11. Cumulative food intake was significantly increased for the duration of the PEP‐064 treatment (Figure 4E). Overall, PEP‐064 treated mice consumed 18% more food than vehicle‐treated controls, and total food intake was higher with PEP‐064 treatment than with anamorelin.

Given the observed changes in body weight, we next examined whether these reflect alterations in body composition. Body composition was assessed by NMR on Day 5 and compared with measurements conducted on Day 19 post‐treatment. Tumour inoculation decreased both fat and lean mass (sham control vs. LLC vehicle) as expected in a cancer cachexia model (Figure 4F). PEP‐064 mitigated the fat losses observed in the model and increased fat mass beyond levels measured in non‐tumour‐bearing mice. As expected, anamorelin also protected against fat mass loss. Neither PEP‐064 nor anamorelin treatment prevented the lean mass losses associated with LLC inoculation. Total leg muscle, heart weight and grip strength were unaffected by LLC inoculation and were not influenced by either of the treatments (Figures 4G–I and S4).

Collectively, these findings further support PEP‐064's potential to restore energy balance in cancer cachexia, driven by its positive effects on food intake, body weight and fat mass.

### 3.4 Whole‐Brain Activity Mapping of PEP‐064 Reveals Neural Correlates Associated With Appetite Control

**FIGURE 5:**
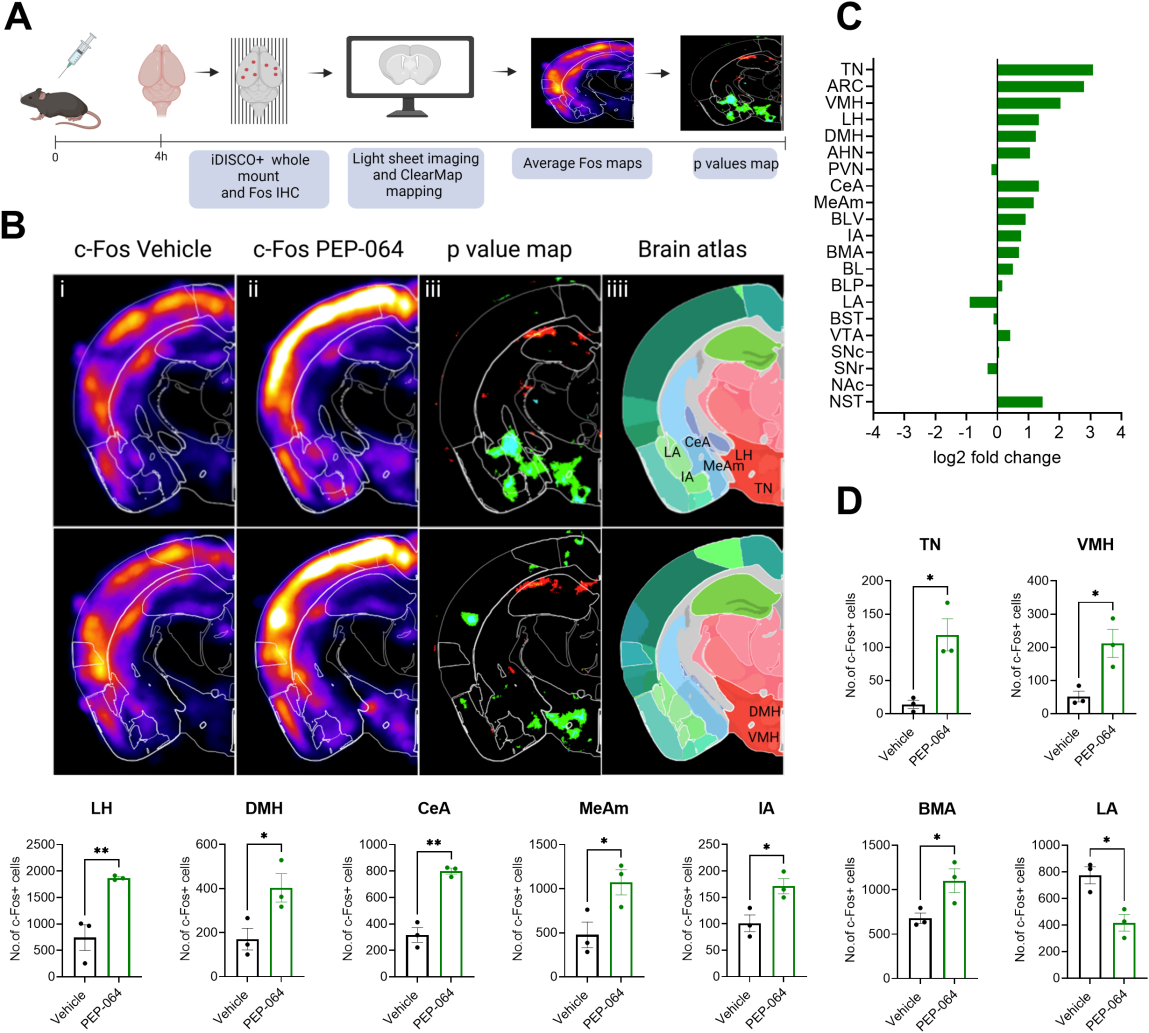
Whole‐brain activity signature in response to PEP‐064. (A) Illustration of the study in C57BL/6 J mice treated with a single s.c. injection of vehicle or PEP‐064 1000 nmol/kg. (B) c‐Fos raw results in two planes after (i) vehicle and (ii) PEP‐064 treatment, (iii) significantly different c‐Fos expression between vehicle and PEP‐064 and (iv) annotated brain atlas of *p*‐value map and significantly differently regulated brain region graphs compared to Vehicle. (C) log2fold changes in c‐Fos expression in common brain areas involved in appetite regulation relative to vehicle. (D) Significantly differently regulated brain regions compared to the vehicle. Data are shown as mean with SEM, *n* = 3. *p < 0.05 and **p < 0.01 analysed by one‐way ANOVA, multiple comparison, Dunnett's post hoc test or unpaired *t* test. AHN, anterior hypothalamic nucleus; ARC, arcuate hypothalamic nucleus; BL, basolateral amygdalar nucleus anterior part; BLP, basolateral amygdalar nucleus; BLV, basolateral amygdalar nucleus ventral part; BMA, basomedial amygdalar nucleus; BST, stria terminalis; CeA, central amygdalar nucleus; DMH, dorsomedial hypothalamus; IA, intercalated amygdalar nucleus; LA, lateral amygdalar nucleus; LH, lateral hypothalamus; MeAm, medial amygdalar nucleus; Nac, nucleus accumbens; NST, nucleus of the solitary tract; PVN, paraventricular hypothalamic nucleus; SNc, substantia nigra pars compacta; SNr, substantia nigra pars reticulata; TN, tuberal nucleus; VMH, ventromedial hypothalamus; VTA, ventral tegmental area.

Given the impact of PEP‐064 on food intake, we sought to identify the neural substrates modulated by its treatment. Because appetite control and body weight regulation are governed by distinct neuronal populations, we conducted a whole‐brain screen for activity‐driven changes using the immediate early gene Fos as a marker, using the unbiased whole‐brain imaging approach iDISCO+ and ClearMap [18] (Figures 5A and S5). By means of deep neuronal networks, we generated a list of brain regions that were more active in mice treated with PEP‐064 over the vehicle group. PEP‐064 increased neuronal activity in critical feeding‐related loci in the hypothalamus (canonical centre for appetite regulation). In brief, there was an increase in c‐Fos expression in the tuberal nucleus (TN), ventromedial hypothalamus (VMH), lateral hypothalamus (LH) and dorsomedial hypothalamus (DMH) compared to vehicle‐treated controls (Figure 5B). The TN was the most affected brain region, exhibiting a threefold increase in activity, followed by a 2.7‐fold increase in the arcuate nucleus (ARC) (Figure 5C). Other areas previously implicated in feeding control also exhibited increased neuronal activity; for instance, most of the amygdalar complex (central amygdalar nucleus [CeA], medial amygdalar nucleus [MeAm], intercalated amygdalar nucleus [IA] and basomedial amygdalar nucleus [BMA]) demonstrated enhanced neuronal activity. In contrast, decreased activity was observed in the lateral amygdalar nucleus (LA) (Figure 5C). Several other regions demonstrated a tendency of increased neuronal activity induced by PEP‐064 treatment (midbrain, basal forebrain and specific brainstem loci) (Figure 5C). Table S1 presents the individual counts of c‐Fos‐positive cells in all brain regions for each mouse. Collectively, the data demonstrate the capacity of PEP‐064 to stimulate hypothalamic neuronal activity associated with appetite control.

### 3.5 PEP‐064 Induces the Secretion of Growth Hormone

**FIGURE 6:**
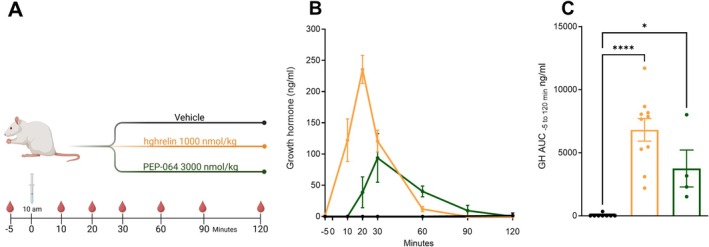
PEP‐064 growth hormone secretion profile in rats. (A) Study outline. (B) Growth hormone secretion following single dosing and (C) area under the curve −5 to 120 min. Data are shown as mean with SEM, *n* = 4–11. *p < 0.05 and ****p < 0.0001 by one‐way ANOVA compared to vehicle.

To examine whether PEP‐064 has the expected effect on GH release, the stimulatory impact of PEP‐064 on GH after a single high s.c. dose was investigated acutely in rats by collecting blood samples between −5 and 120 min and comparing the results to hghrelin (1000 nmol/kg) as a positive control (Figure 6A). As anticipated, PEP‐064 treatment increased GH secretion compared to the vehicle (Figure 6B,C), with levels peaking at 93 ng/mL at 30 min and gradually returning to near baseline by 120 min. Contrastingly, hghrelin rapidly stimulated GH secretion, peaking at 20 min at 235 nmol/kg and rapidly returned to baseline at around 90 min. Taken together, these results demonstrate that PEP‐064 acutely stimulates GH release in rats, albeit with a lower magnitude and slower kinetics compared to the robust and rapid response induced by hghrelin.

## 4 Discussion

Cancer cachexia is a debilitating syndrome that remains underdiagnosed and undertreated, with a lack of efficacious and approved drugs. In this paper, we present PEP‐064, a novel half‐life extended ghrelin analogue for treating cancer cachexia. We demonstrate that lipidation and backbone stabilization of the native ghrelin peptide resulted in an efficacious peptide that induced hyperphagia and promoted pronounced body weight gain in healthy mice. Furthermore, PEP‐064 restored energy balance in preclinical cancer cachexia models by increasing food intake and body weight, preserving fat and lean mass and increasing adiposity. Mechanistically, we show peripheral PEP‐064 administration activates key appetite‐related loci in the hypothalamus and stimulates GH secretion.

In vitro characterization identified PEP‐064 as a promising lead candidate with an extended plasma half‐life of 6.6 h versus 10 min for native ghrelin. In healthy mice, PEP‐064 demonstrated superior efficacy in stimulating food intake, body weight and adiposity compared to native ghrelin, with no evidence of desensitization, indicating that the lipidation and stabilization resulted in a constitutively active ghrelin peptide. Furthermore, the doses employed throughout this study ranged from 100–3000 nmol/kg with no observable adverse effects, supporting the tolerability and safety profile of PEP‐064.

Given the highlighted potential of ghrelin‐based therapeutics in cancer cachexia, we sought to profile PEP‐064 in two preclinical models: the C26 colon and LLC model. In our hands, these models presented a moderate cachexia phenotype due to ethical considerations of tumour endpoint (C26) and the choice of cell line (LLC), which limited examining PEP‐064 in the full progression of cachexia, especially in the context of skeletal muscle wastage. However, cachexia uses a scaling definition that progresses from precachexia to cachexia, and further to refractory cachexia. Although refractory cachexia presents with the classical hallmarks of cachexia, namely, extreme weight loss with both fat and muscle wastage, it is end‐stage and unresponsive to treatment, and a rational target for drug intervention should be available at moderate levels of cachexia, as seen in our models [19].

Patients with cancer cachexia experience reduced appetite, and anorexia eventually develops in most patients [20]. Our models lacked anorexia, yet PEP‐064 treatment increased total food intake by approximately 18% in both our LLC and C26 cancer cachexia models. These findings corroborate ghrelin responsiveness being preserved in cancer cachexia, contrary to the proposed concept of ghrelin resistance [21, 22]. However, in the LLC model, PEP‐064 increased food intake during the first 8 days, returning to baseline on Days 11–14. Rather than showing receptor desensitization, which was not observed in either the healthy mice or the C26 model, we speculate that the exceptionally large tumour burden in the LLC mice increased the metabolic demands and nonspecific pathological effects associated with tumour growth to a degree that overrode the upregulated appetitive signalling. Similar findings in the anamorelin positive control group further support the conclusion that this effect was driven by extreme tumour burden. Together, the results demonstrate PEP‐064's ability to positively influence nutritional status, which is a major aim of anticancer cachexia treatments to improve treatment success, quality of life and survival rate.

In cancer cachexia, when weight loss exceeds 5%, the risk of patient mortality is greatly increased [23] and linked to poor anticancer therapeutic outcomes [24], emphasizing the critical importance of maintaining body weight as a primary goal in oncologic strategies. In both the C26 and LLC preclinical cancer cachexia models, PEP‐064 ameliorated the tumour‐associated body weight loss, without increasing tumour weight. In the LLC model, although food intake was maintained, the ability of PEP‐064 to preserve body weight diminished at very high tumour burdens, likely due to the increased metabolic demands imposed by tumour growth. Body composition analysis by MRI and measured fat depots showed that PEP‐064 provided robust protection from tumour‐associated fat mass losses. This is clinically relevant in cancer cachexia, as a higher body weight before treatment or diagnosis has been suggested to mitigate its severity. This is based on the ‘obesity paradox’ hypothesis, which proposes that having greater body reserves may offer a protective effect and aid energy balance [25, 26]. Furthermore, fat mass acts as a reservoir that can serve as a potential energy source, and adipose tissue secretes anti‐inflammatory and metabolically protective adipokines such as adiponectin, apelin and omentin. Adiponectin is notably reduced in patients with cancer cachexia, and restoration of its signalling has shown beneficial effects in preclinical cancer cachexia models [27].

In addition to its beneficial effects on fat mass, PEP‐064 protected from tumour‐driven losses in lean mass in the C26 model. Similar, positive lean mass effects were seen in the LLC model utilizing the long‐acting ghrelin analogue EXT‐418, suggesting that long‐acting ghrelin analogues have a strong potential to improve overall body composition [17]. Given the lack of GHRS‐1α receptors on skeletal muscle, PEP‐064's protective effect on lean mass suggests the involvement of either GHRS‐1α‐independent mechanisms or secondary mediators [28]. Here, we investigated PEP‐064's potential to stimulate GH, which has been shown to regulate protein anabolism via IGF‐1‐dependent endocrine and paracrine mechanisms as well as IGF‐1‐independent pathways [29]. Indeed, we demonstrate that PEP‐064 robustly stimulates GH secretion, which could be a potential mechanism through which PEP‐064 could exert a positive influence on lean mass over time; however, we did not reconfirm GH secretion in the C26 or LLC study. Direct effects on skeletal muscle were evaluated through measurements of muscle weight, CSA and grip strength testing. Across all assessments, there was no clear evidence of overt muscle wasting in either model. However, a consistent, non‐significant trend towards reduced values was observed following tumour inoculation, suggesting that both models reflect a moderate stage of cachexia with limited muscle loss. Consequently, the ability to detect muscle‐specific rescue effects of PEP‐064 was constrained by a lack of baseline phenotype. Nevertheless, under our experimental conditions, PEP‐064 treatment did not significantly alter any direct muscle parameters. Taken together, these findings suggest that if there were any rescue effects of PEP‐064 on skeletal muscle, they are likely modest and much less pronounced than its effects on adipose tissue. In this context, combining PEP‐064 with anabolic or muscle‐targeted therapies may represent a promising strategy for achieving more comprehensive protection against cancer cachexia.

To unbiasedly comprehend which neural correlates are responsible for PEP‐064's mode of action on food intake, we performed a whole‐brain screen of the expression of the immediate early gene Fos. PEP‐064's c‐Fos immunoreactivity could be mapped to hypothalamic regions where the highest density of ghrelin receptors in the brain is located [30]. Our results are in line with other studies in rodents utilizing native ghrelin [3] and synthetic ghrelin agonists; however, less activation was quantified in the brainstem in our study compared to other groups [31, 32, 33]. Interestingly, the highest fold change in activity was found in an understudied hypothalamic region, the TN. Although knowledge about the TN remains scarce, it has been previously reported to play a role in energy homeostasis and appetite, involving a distinct neuronal subtype, the somatostatin‐positive neurons (^TN^SST neurons) [34]. Relevant to our work, ^TN^SST neurons express GHSR; furthermore, ghrelin treatment increased c‐Fos immunoreactivity and increased the baseline firing of ^TN^SST neurons in situ. Additionally, activation of ^TN^SST neurons increased food intake, whereas inhibition decreased feeding time and frequency through ascending projections to the PVN and bed nucleus of the stria terminalis. Given the demonstrated potential of ghrelin to increase food intake via TN activation, it could be hypothesized that the PEP‐064 stimulatory effect on feeding could be driven by TN neuronal activation; however, future studies are required to resolve the precise circuitry responsible for such a response. In addition to its role in the homeostatic regulation of energy balance, ghrelin also contributes to the hedonic component of appetite by activating opioid and dopamine receptors [35]. This interaction amplifies the rewarding and motivational reactions to food cues, which could be another unique mechanistic way of combating cachexia‐associated anorexia and the decreased QoL surrounding decreased appetite in patients with cancer cachexia. Indeed, PEP‐064 increased VTA activation, a canonical centre in the dopaminergic reward circuitry. Other regions of interest with marked increases in activity were located in the amygdala complex, in particular the CeA, which plays a role in assessing the valence and salience of learned food cues. In summary, our findings align with published literature and argue for PEP‐064's ability to target both homeostatic and hedonic appetitive responses, ultimately leading to an increase in food intake.

This study is subject to several limitations. First, only male mice were used in the studies; therefore, we have no understanding of the sex‐mediated differences in PEP‐064 treatment. Second, our use of young mice in the studies, although cancer cachexia is linked to aged populations, may not have captured the interplay between cancer cachexia and sarcopenia, which would be an important consideration when assessing muscle atrophy. Third, although commonly used, the preclinical cancer cachexia models used do not fully reflect human disease. They are aggressive but lack severe weight loss, skeletal muscle wastage and tumour treatment, and their shorter timelines may underestimate treatment benefits compared to longer clinical courses. To comprehensively assess the therapeutic potential of PEP‐064 in cancer cachexia, future studies should incorporate longer duration cachexia models, such as orthotopic models, along with anticancer combination therapies, to better reflect the heterogeneity of cachexia in cancer patients [36].

In conclusion, PEP‐064 restored energy balance in cancer cachexia by increasing food intake and body weight, preserving lean mass and increasing adiposity, without affecting tumour growth. Considering the unmet medical need for safe and effective treatments for cachexia, our study highlights the feasibility of a stabilized, half‐life‐extended ghrelin peptide approach and revitalizes the potential of ghrelin‐based therapies for cancer cachexia.

## Funding

This work was supported by the National Institute of Diabetes and Digestive and Kidney Diseases (R00DK1208689 and P30DK045735), the National Institute of Health Yale‐Murine Tissue Mapping Center (U54 AG079759), the National Institute of Health National Institutes of Aging (P30AG066508) and the Innovation Fund (INF) Denmark (2041‐00003B, 3130‐00004B and 3146‐00040B).

## Ethics Statement

The authors certify that they gave their informed consent prior to their inclusion in the study and that the work complies with the ethical guidelines for authorship and publishing in the Journal of Cachexia, Sarcopenia and Muscle. All animal experiments were performed in accordance with the ethical standards laid down in the 1964 Declaration of Helsinki and its later amendments. The manuscript does not contain clinical studies or patient data.

## Conflicts of Interest

The authors declare no conflicts of interest.

## Supporting information


**Table S1:** Individual counts of c‐Fos‐positive cells in all brain regions for each mouse.

**Figure S1:** Representative images of Hematoxylin and eosin (H&E) stained soleus muscle tissue and muscle fiber cross‐sectional area quantification on whole‐section images using Cellpose extension in QuPath. Three sections per animal were analysed and averaged.
**Figure S2:** (A) In vitro signaling profile of PEP‐064 on the mouse and (B) human GHSR‐1α. (C) PEP‐064 pharmacokinetics following subcutaneous s.c. administration of 300 nmol/kg.
**Figure S3:** Wet muscle weights in C26 colon carcinoma mouse model of cancer cachexia. (A) gastrocnemius, (B) soleus, (C) tibialis anterior (D) extensor digitorum longus, and quadriceps. Data are shown as mean with SEM, *n* = 8–11, by One‐way ANOVA compared to C26 + Vehicle.
**Figure S4:** (A) Maximum grip strength, defined as the highest value from 3 trials performed on day 19. (B) Change in maximum grip strength from day 5 to day 19. (C) Change in average grip strength (mean of 3 trials) from day 5 to day 19. Data are presented as mean ± SEM, *n* = 8–12.
**Figure S5:** (A) Primary data of c‐Fos labeling in the whole brain, (i) Allen Brain Atlas depicting brain regions, (ii) *p*‐value map showing the significantly different c‐Fos expression between vehicle and PEP‐064 treated mice, (iii) heatmap of c‐Fos protein distribution and raw c‐Fos data in vehicle and (iiii) PEP‐064 treated mice. (B) Primary data of c‐Fos labeling in the Tuberal Nucleus (TN). (i) Allen brain atlas annotation of the TN and corresponding images in Fos channel of the (i) vehicle and (ii) PEP‐064 treated mice. 2D images correspond to one representative animal from each group, image colours were inverted in Fiji for easier visualization.

## Data Availability

The data that support the findings of this study are available from the corresponding author upon reasonable request.
