# Supplementary material for: Novel Long‐Acting Ghrelin Analogue PEP‐064 Restores Energy Balance in C26 and Lewis Lung Carcinoma–Induced cachexia in Mice
Source: J Cachexia Sarcopenia Muscle. 2026 Jun 1;17(3):e70318. doi: 10.1002/jcsm.70318 (PMC13240157; doi:10.1002/jcsm.70318)
Supplement: Supplementary file 2 — Figure S1: Representative images of Hematoxylin and eosin (H&E) stained soleus muscle tissue and muscle fiber cross‐sectional area quantification on whole‐section images using Cellpose extension in QuPath. Three sections per animal were analysed and averaged. Figure S2: (A) In vitro signaling profile of PEP‐064 on the mouse and (B) human GHSR‐1α. (C) PEP‐064 pharmacokinetics following subcutaneous s.c. administration of 300 nmol/kg. Figure S3: Wet muscle weights in C26 colon carcinoma mouse model of cancer cachexia. (A) gastrocnemius, (B) soleus, (C) tibialis anterior (D) extensor digitorum longus, and quadriceps. Data are shown as mean with SEM, n = 8–11, by One‐way ANOVA compared to C26 + Vehicle. Figure S4: (A) Maximum grip strength, defined as the highest value from 3 trials performed on day 19. (B) Change in maximum grip strength from day 5 to day 19. (C) Change in average grip strength (mean of 3 trials) from day 5 to day 19. Data are presented as mean ± SEM, n = 8–12. Figure S5: (A) Primary data of c‐Fos labeling in the whole brain, (i) Allen Brain Atlas depicting brain regions, (ii) p‐value map showing the significantly different c‐Fos expression between vehicle and PEP‐064 treated mice, (iii) heatmap of c‐Fos protein distribution and raw c‐Fos data in vehicle and (iiii) PEP‐064 treated mice. (B) Primary data of c‐Fos labeling in the Tuberal Nucleus (TN). (i) Allen brain atlas annotation of the TN and corresponding images in Fos channel of the (i) vehicle and (ii) PEP‐064 treated mice. 2D images correspond to one representative animal from each group, image colours were inverted in Fiji for easier visualization. [file JCSM-17-e70318-s002.docx]

**Supplementary data**

*Western blot method*

Muscle tissue was pulverized in liquid nitrogen before being homogenized in a modified GSK3-buffer (10% glycerol, 1% NP-40, 20 mM sodium pyrophosphate, 150 mM sodium chloride (NaCl), 50 mM HEPES (pH 7.5), 20 mM β-glycerophosphate, 10 mM sodium fluoride (NaF), 2 mM phenylmethylsulfonyl fluoride (PMSF), 1 mM EDTA (pH 8.0), 1 mM EGTA (pH 8.0), 2 mM Na3VO4, 10 μg/mL leupeptin, 10 μg/mL aprotinin, 3 mM benzamidine). The homogenization was performed using a Tissue-Lyser II with stainless steel grinding balls (2 × 30s at 30 Hz) (Qiagen, USA). After 30 min of end-over-end at 4 °C, the samples were centrifuged at 9.500 g for 20 min at 4 °C. The supernatant was collected, discarding the remaining pellet.

Lysate protein concentration was determined using the bicinchoninic acid method. Bovine serum albumin (BSA) was used as a standard (Pierce). Immunoblotting of relevant phosphorylation sites of proteins, as well as total proteins, was performed by standard immunoblotting techniques using commercially available equipment (BioRad Laboratories, USA). After the transfer of protein to polyvinylidene difluoride membranes, these were subsequently blocked for 5 min in TBS-Tween 20 containing either 2 % skim milk or 3 % BSA at room temperature. The membranes were incubated overnight with primary antibodies at 4 °C. On the following day, the primary antibody was removed, membranes washed 2-3 times for 5 minutes in TBS-Tween 20, after which horseradish peroxidase-conjugated secondary antibody was applied (1:5000) and incubated for 45 min at room temperature. After 2-3 washes of 5-10 minutes with TBS-Tween 20, imaging and visualization of bands were performed using Bio-Rad ChemiDocTM MP Imaging System and enhanced chemiluminescence (ECL+; Amersham Biosciences). All blots were quantified using Coomassie as a loading control.

| **Target** | **Primary Antibody** | **Dilution** |
| --- | --- | --- |
| MuRF-1 | Santa cruz #398608 | 1:1000 |
| Atrogin-1 | Santa cruz #166806 | 1:1000 |
| Akt S473 | Cell signaling #9271 | 1:1000 |
| Akt | Cell signaling #3063 | 1:1000 |
| Stat 3-p | Santa cruz # 9145 | 1:1000 |
| Stat 3 | Santa cruz # 4904 | 1:1000 |

**Supplementary figure 1**


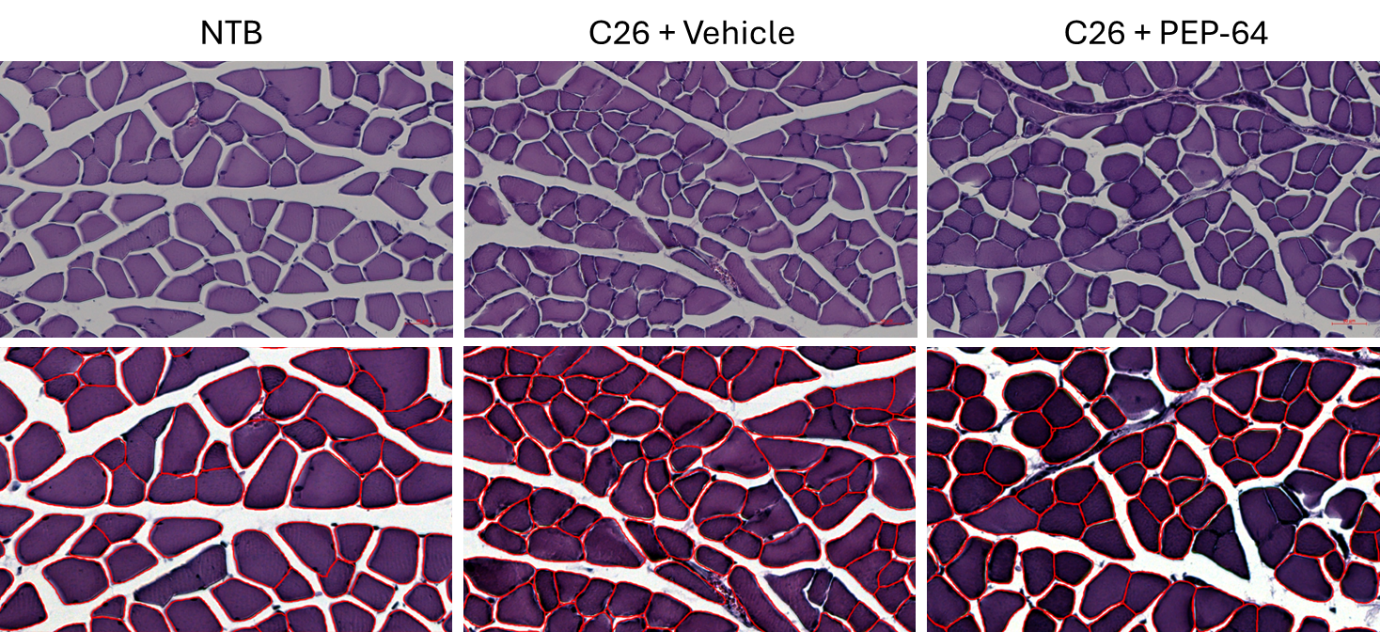


C26 + PEP-064

C26 + Vehicle

NTB

**Supplementary figure 1.** Representative images of Hematoxylin and eosin (H&E) stained soleus muscle tissue and muscle fiber cross-sectional area quantification on whole-section images using Cellpose extension in QuPath. Three sections per animal were analyzed and averaged.

**Supplementary figure 2**

**
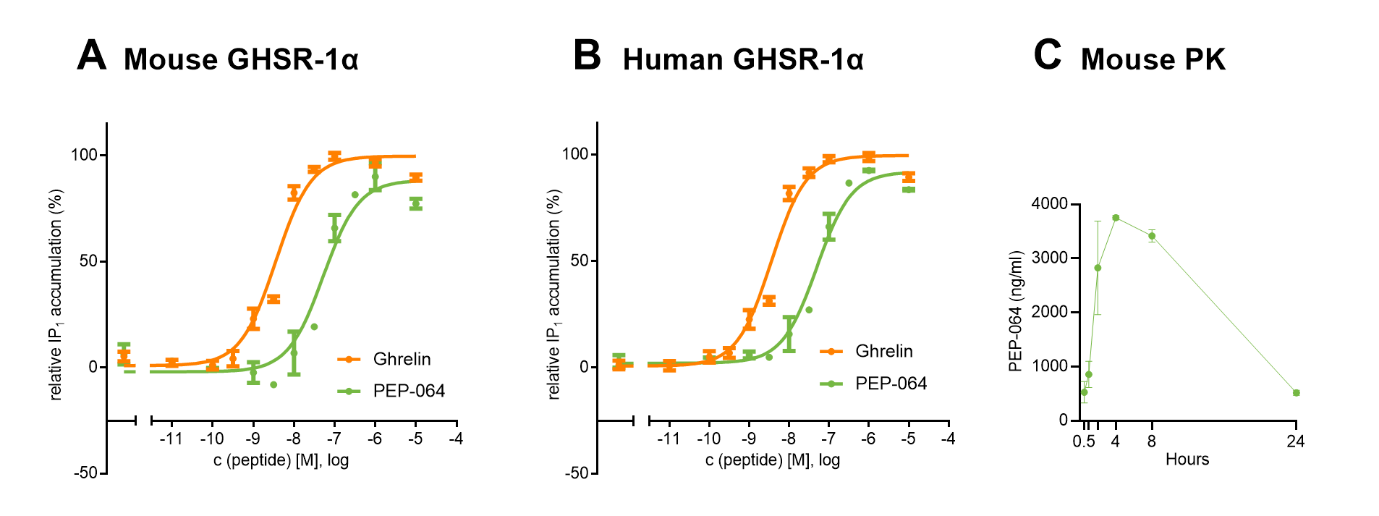
**

**Supplementary figure 2**. (A) In vitro signaling profile of PEP-064 on the mouse and (B) human GHSR-1α. (C) PEP-064 pharmacokinetics following subcutaneous s.c. administration of 300 nmol/kg.

**Supplementary figure 3**
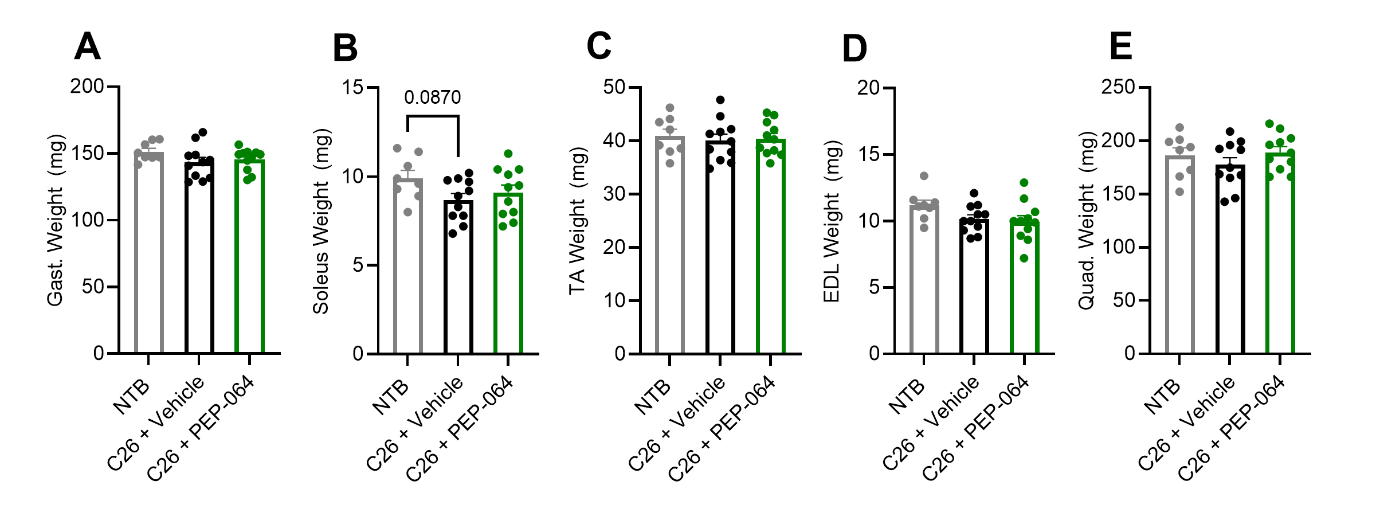
**Supplementary figure 3**. Wet muscle weights in C26 colon carcinoma mouse model of cancer cachexia. (A) gastrocnemius, (B) soleus, (C) tibialis anterior (D) extensor digitorum longus, and quadriceps. Data are shown as mean with SEM, n=8-11, by One-way ANOVA compared to C26 + Vehicle.

**Supplementary figure 4**

**
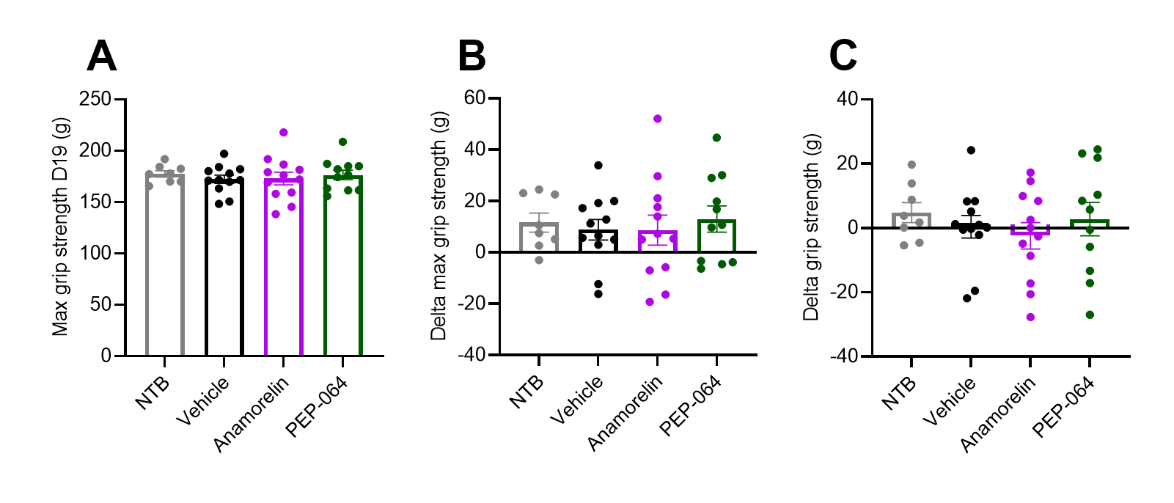
**

**Supplementary figure 4**. (A) Maximum grip strength, defined as the highest value from 3 trials performed on day 19. (B) Change in maximum grip strength from day 5 to day 19. (C) Change in average grip strength (mean of 3 trials) from day 5 to day 19. Data are presented as mean ± SEM, n=8-12.

**Supplementary figure 5**

**
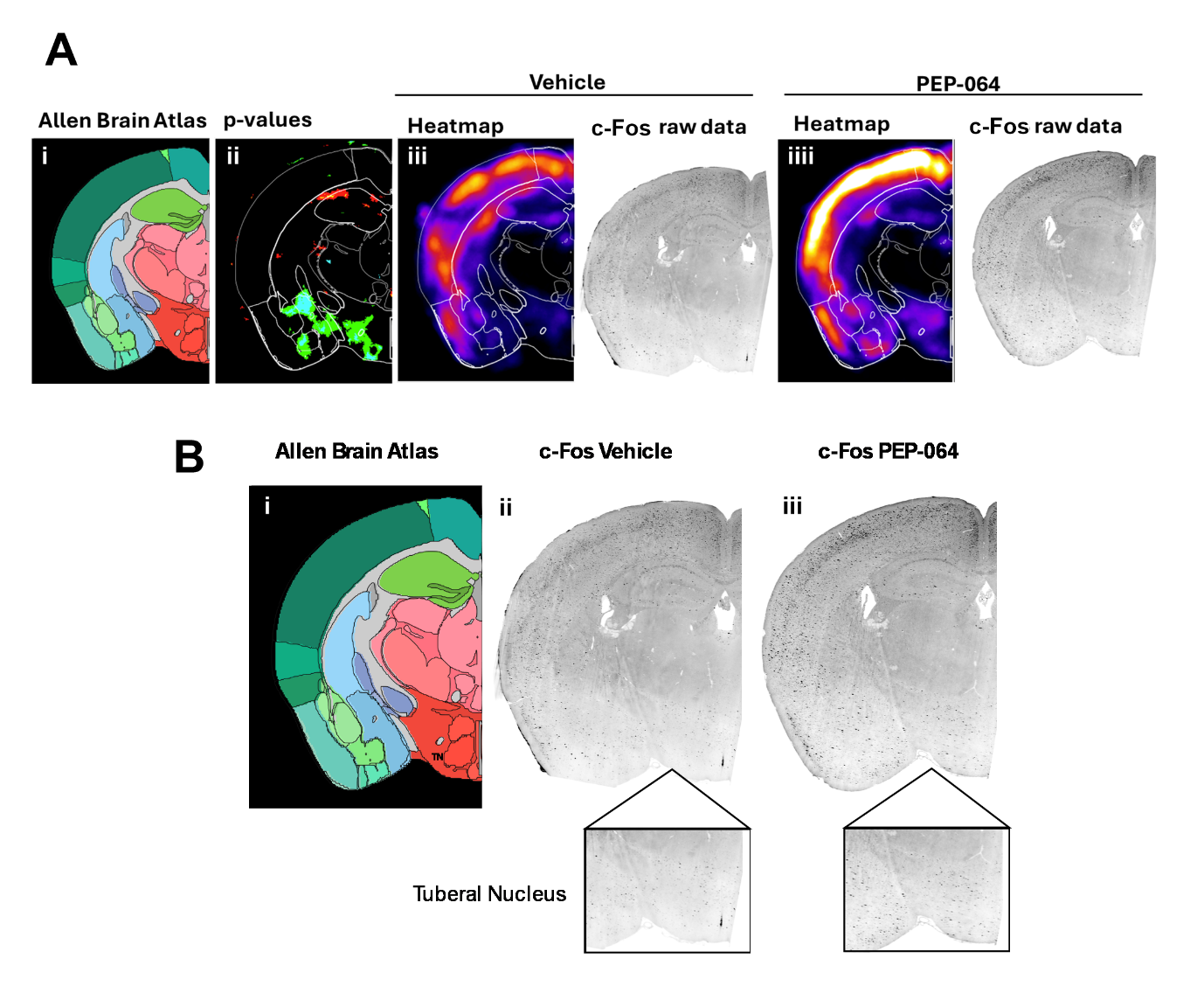
**

**Supplementary figure 5.** (A) Primary data of c-Fos labeling in the whole brain, (i) Allen Brain Atlas depicting brain regions, (ii) p-value map showing the significantly different c-Fos expression between vehicle and PEP-064 treated mice, (iii) heatmap of c-Fos protein distribution and raw c-Fos data in vehicle and (iiii) PEP-064 treated mice. (B) Primary data of c-Fos labeling in the Tuberal Nucleus (TN). (i) Allen brain atlas annotation of the TN and corresponding images in Fos channel of the (i) vehicle and (ii) PEP-064 treated mice. 2D images correspond to one representative animal from each group, image colors were inverted in Fiji for easier visualization.
